# Spatial RNA sequencing methods show high resolution of single cell in cancer metastasis and the formation of tumor microenvironment

**DOI:** 10.1042/BSR20221680

**Published:** 2023-02-23

**Authors:** Yue Zheng, Xiaofeng Yang

**Affiliations:** 1 Department of Biochemistry and Molecular Biology, Basic Medical College, Shanxi Medical University, No. 56, Xinjiang South Road, Yingze street, Yingze District, Taiyuan City, Shanxi Province 030000, China; 2 Department of Urology, First Hospital of Shanxi Medical University, No. 85, Jiefang South Road, Yingze street, Yingze District, Taiyuan City, Shanxi Province 030000, China

**Keywords:** cancer metastasis, in situ RNA sequencing, multimodal analysis, single cell RNA sequencing, spatial transcriptomics, tumor microenvironments

## Abstract

Cancer metastasis often leads to death and therapeutic resistance. This process involves the participation of a variety of cell components, especially cellular and intercellular communications in the tumor microenvironment (TME). Using genetic sequencing technology to comprehensively characterize the tumor and TME is therefore key to understanding metastasis and therapeutic resistance. The use of spatial transcriptome sequencing enables the localization of gene expressions and cell activities in tissue sections. By examining the localization change as well as gene expression of these cells, it is possible to characterize the progress of tumor metastasis and TME formation. With improvements of this technology, spatial transcriptome sequencing technology has been extended from local regions to whole tissues, and from single sequencing technology to multimodal analysis combined with a variety of datasets. This has enabled the detection of every single cell in tissue slides, with high resolution, to provide more accurate predictive information for tumor treatments. In this review, we summarize the results of recent studies dealing with new multimodal methods and spatial transcriptome sequencing methods in tumors to illustrate recent developments in the imaging resolution of micro-tissues.

## Introduction

The early stage of cancer is usually considered curable using surgical resection and therapeutic agents [1]. Treatment of local cancer tissues therefore results in good survival, while metastatic cancer is difficult to treat by surgical resection and usually results in therapeutic resistance [2]. This may be because cancer metastasis involves multiple organs and tissues in the body and is therefore a complex and variable system. In recent years, research on the tumor microenvironment (TME) during tumor metastasis has been limited to solid cancer tissues, and has involved the tissue environment that affects its growth and proliferation [3]. We therefore need to fully understand the inherent genetic information of tumor cells and the TME involved in its survival to better understand the development of tumors from initiation to metastasis and to find effective therapies.

**Figure 1:**
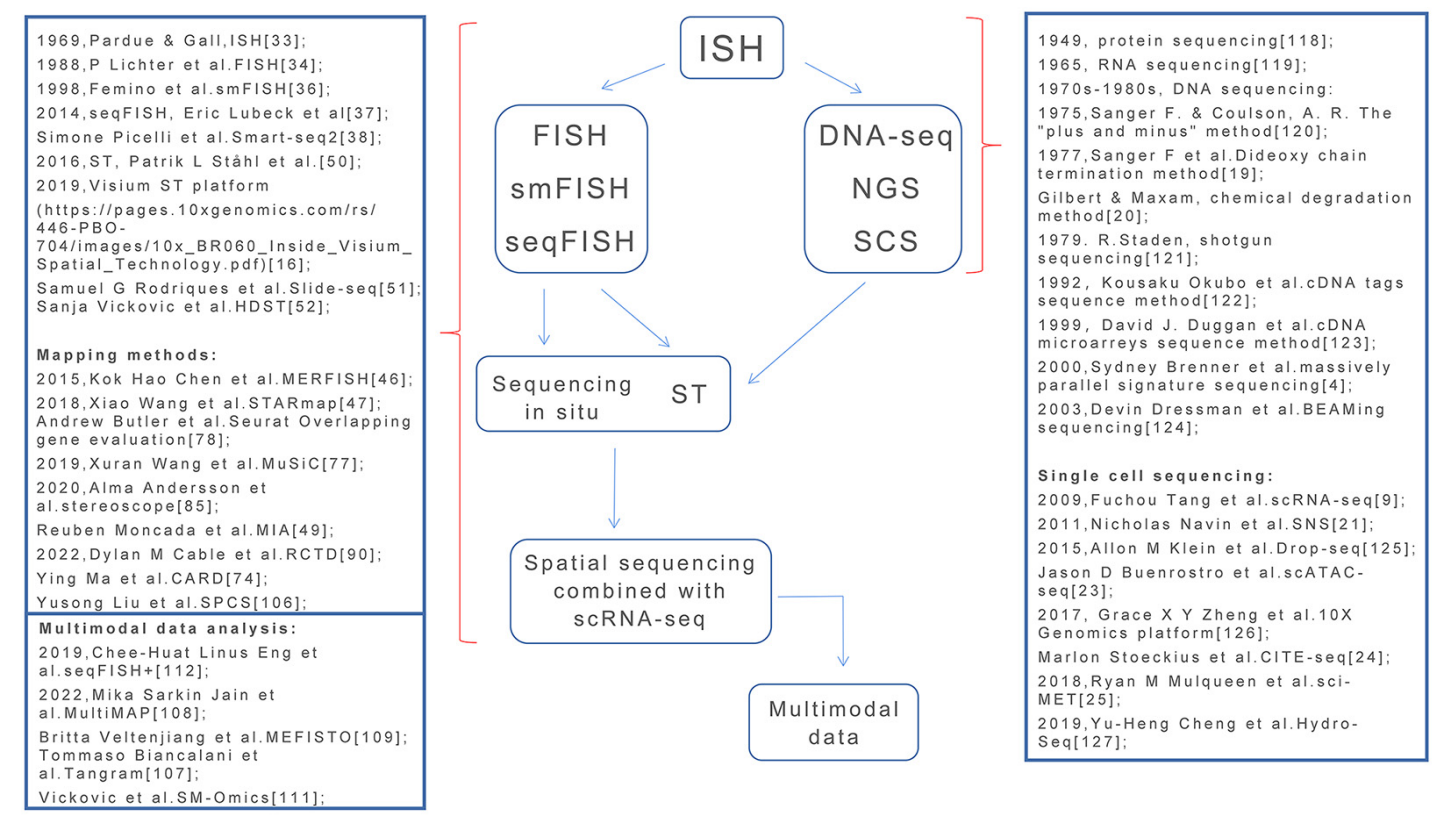
A diagram of the development of the main sequencing methods The development of sequencing methods, from molecular hybridization to high-throughput RNA sequencing of a single cell, and the integration of multimodal datasets of spatial maps.

The next-generation sequencing (NGS) has the advantages of large-scale and high-throughput gene sequencing [4] (Figure 1). Researchers have completed the human genome project (HGP) [5], revealing the normal genome characteristics of humans, and have promoted research on establishing a gene atlas of human diseases and cancers [6].

However, NGS has been largely directed toward sequencing malignant genes, which has mainly ignored important information of non-malignant samples involved in cancer cell progression in the TME [7]. Moreover, NGS has mainly been directed to sequencing the mutant genes in whole tissues [8] and has been unable to distinguish gene mutations belonging to specific cell types. As a result, we do not know whether these mutations originated from primary or metastatic cancers, or from free cancer cells during the process of metastasis.

The subsequent use of single-cell RNA sequencing (scRNA-seq) has largely compensated for the aforementioned deficiency [9]. NGS mainly involves detection of mutant gene expressions, biological functions, and signal pathways in whole tissues. The above three processes can then be classified into different cell populations to reveal the evolutionary process of cells with cancer-related mutant genes [10], from the formation of primitive cancer stem cells [11] to the development of primary cancer lesions, and then to the diffusion of other tissues, organs or fluids [12,13]. In addition, using mutant genes identified in previous studies, we can now determine which cells belong to cancer cells and which cells provide help for the proliferation of tumor cells [14], and we can identify mutant genes expressed by some rare cell subsets, for a deeper understanding of the roles of tumor cells [15].

Due to the inability to detect whole tissue sections, important information between cells and tissues may be omitted [16]. However, combining data with subsequent spatial transcriptome sequencing technology can intuitively reflect changes of cell types [17], the locations of cell populations in the TME, and their interactions with cancer cells during metastasis [18]. By combining multiomics data with spatial sequencing, characterization of single cells is now possible, with subsequent technologies to examine whole tissue components that previous methods could not detect.

In this review, we summarize studies on the use of spatial transcriptome technologies to characterize tumor metastasis and the TME, and describe future multimodal analysis methods that combine multiomics and multidimensional methods in spatial transcriptome technology.

## Results

### The development and limitations of scRNA-seq

The use of scRNA-seq, first used in 2009, successfully detected multiple transcriptional variants expressed in the same cell [9]. To provide a more accurate classification and more information of rare transcriptional information in samples, which was not possible using NGS, scRNA-seq was used for sequencing mutant genes in whole tissues.

Just as the initial DNA sequencing method of Sanger and Gilbert led to genetic sequencing [19,20], scRNA-seq led to single-cell sequencing (SCS). Some significant SCS methods involve the following: single-nucleus RNA sequencing [21], single-cell DNA sequencing (scDNA-seq) [22], and single-cell ATAC-seq (the accessible genome of individual cells by assaying for transposase-accessible chromatin using sequencing, ATAC-seq) (scATAC-seq) [23], which are combined with scDNA-seq to detect chromatin. Cellular indexing of transcriptomes and epitopes by sequencing (CITE-seq) [24] is combined with scRNA-seq to detect proteins. Single-cell combinatorial indexing for methylation analysis (sci-MET) [25] has also been combined with scDNA-seq to detect methylation (Figure 1). Other technologies and the main development of SCS are in Figure 1[118–127].

For tumor cells with high intratumoral heterogeneities (ITHs) [26], single cell genome sequencing can analyze the evolution process of genetic material in the tumors to identify internal factors leading to the formation of tumor cells. However, formation and metastasis of tumors do not involve a single process, but result in interactions with other adjacent non-malignant cells, to promote the development of tumors, in a process involving the TME [27]. Although it is not possible to identify cell types participating in the TME and epithelial–mesenchymal transformation (EMT) processes using scDNA-seq, it is now possible to achieve this goal during cancer metastasis and in the TME using transcriptome sequencing of single cells.

However, a tumor is not independent of the rest of the body. Like other tissues and organs, it depends on the overall regulation system of the body during its formation. For example, the abnormal function of the extracellular matrix in the TME causes cancerous epithelial cells to lose polarity and adhesion, change morphology, and move to surrounding tissues [28].

While scRNA-seq separates the whole tissue sample into individual cells by analyzing a cell suspension, it loses the whole system of interactions with the body after *in vitro* dissociation, therefore, scRNA-seq studies are limited to independent cell populations [29]. For complex and variable mechanisms of the TME and cancer metastasis, we can only infer and verify the possible mechanisms using specific mutant genes and cell phenotypes. Regarding how the TME and cancer metastasis proceed, we still do not have a comprehensive, systematic, and deep understanding. Thus, scRNA-seq has limitations in reflecting the integrity of tumor tissues.

In addition, scRNA-seq itself has some technical limitations, including that freezing and dissociation experimental steps may lead to partial cell death [30]. The variable transcription of eukaryotic cells causes noise in sequencing [31], and corrections of batch effects and the ectopic gene expressions may lead to inaccurate identifications of cell subsets [32]. In conclusion, to understand the mechanism of tumor metastasis and the TME more comprehensively, we need better methods.

### *In situ* spatial transcriptomic sequencing

In 1969, Pardue and Gall developed *in situ* hybridization (ISH) [33], using nucleotide probes to detect the sequence of mutant genes within cells. This was followed by fluorescence *in situ* hybridization (FISH) using fluorescent probes to detect chromosome translocations [34] (Figure 1). In subsequent developments, more accurate and efficient FISH-based technologies have been reported. Delong et al. used multiple probes at the same time to identify multiple cell types in a developmental system of single microbial cells [35]. Femino et al. improved FISH and used a digital imaging microscope to detect single RNA molecules (smFISH), to quantitate RNAs produced by a single cell [36].

However, the above techniques only selected known molecular probes for qualitative and quantitative determinations, and positioning of specific transcriptome information. There are many unknown RNAs in a single cell, which limits detection of the whole transcriptome in cells. Lubeck et al. therefore developed seqFISH [37], using bar codes encoded by fluorescent labeled bases, to combine all possible mRNAs in a single cell, resulting in images showing fluorescent spots marked by different bar codes. Each spot contains cDNAs produced by mRNAs hybridized with bar codes, followed by sequencing of these cDNAs. This method is an expanded version of smFISH, involving hybridizing all possible mRNAs in a single cell at the same time, to detect mRNA sequences of the entire transcriptome of a single cell (Figure 1).

The better the detection of genetic material in a single cell, the higher the localized imaging resolution of a single cell. The emergence of seqFISH has improved sequencing efficiency and cell imaging resolution. Next, Smart-seq2 continues to optimize detection of the single cell transcriptome, focusing on full-length coverage of transcripts, to improve cDNA production and expand establishment of a cDNA library [38] (Figure 1). Except for detection of the transcriptome in a single cell, *in situ* sequencing (ISS) is able to perform targeted detection of short-stranded RNA fragments in tissues with cells, to characterize the effects of cell–cell interactions in tissues on a single cell [39].

The above methods are all directed to the detection of mRNAs. Although long-chain noncoding RNAs (LncRNAs) in cells are not involved in protein coding, they also play an important role in regulating transcription and the activity of proteins [40]. Lv et al. developed LncSpA to produce a spatial map of LncRNA in normal and cancer tissues of humans [41]. Subsequently, Alon et al. developed untargeted expansion sequencing, which expanded the detection of RNA to the entire region, whether it was the targeted or untargeted region, and was able to identify thousands of gene sequences, including spliceosomes, to reveal the tissue localization of tumor cells and immune cells in mouse brain neurons and human metastatic breast cancers, as well as the nano resolution of intracellular RNAs [42].

### Imagining methods of *in situ* spatial transcriptomic sequencing

The fluorescence microscope has been used to image cells in tissue sections, involving laser capture microdissection (LCM) [43], digital imaging microscopy [44], and super-resolution microscopy (SRM) [45]. These methods maintained the imaging flexibility of optical microscopy, by extending the diffraction wavelength, to observe cell substructures.

Since then, SRM-based imaging technologies have been used in numerous studies. Chen et al. developed multiple error robust fluorescence *in situ* hybridization (MERFISH) technology, which could improve sequencing efficiency and minimize errors at the same time, to expand imaging of nearly 1000 different RNAs in hundreds of single cells, to achieve simultaneous improvement of both the efficiency and accuracy [46]. Wang et al. developed spatially resolved transcription amplification readout mapping (STARmap), using a three-dimensional map to obtain *in situ* sequencing of transcriptome information of a single cell, as well as to amplify the target signal [47]. For thicker tissue slices, this technique can also show the distribution gradient of molecules. Furthermore, Jamalzadeh et al. developed QuantISH [48] and used it to characterize high-grade serous carcinomas.

Improvements of molecular probes and imaging technologies are the main factors in improving the accuracy and efficiency of *in situ* spatial transcriptome sequencing. The finer the classification of molecular probes, the more RNAs that can be detected. The higher the imaging resolution of these detection methods of tissue sections, the less the error between detection information and imaging, to achieve a higher degree of accuracy.

In addition, with the improvement of the number and accuracy of *in situ* detection of transcriptomes within a single cell, investigators are no longer limited to studies of a single isolated cell, but can identify the influence between cells and the tissue environment, to evaluate the process of the transcriptome of the inner cells more comprehensively.

**Figure 2:**
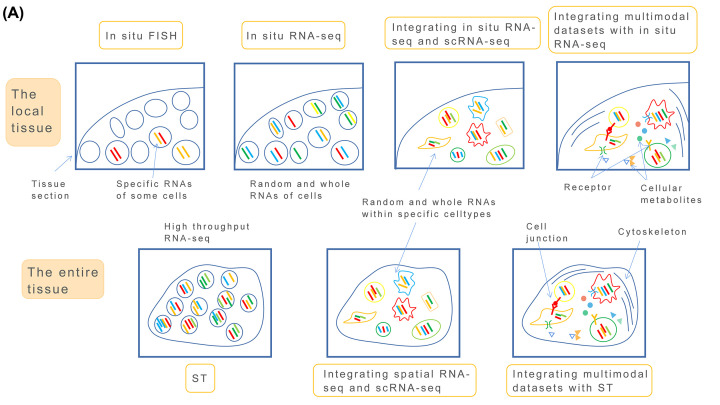
A diagram shows the differences between in situ and ST spatial RNA sequencing The difference of *in situ* and ST spatial RNA sequencing (**A**).

**Figure 3:**
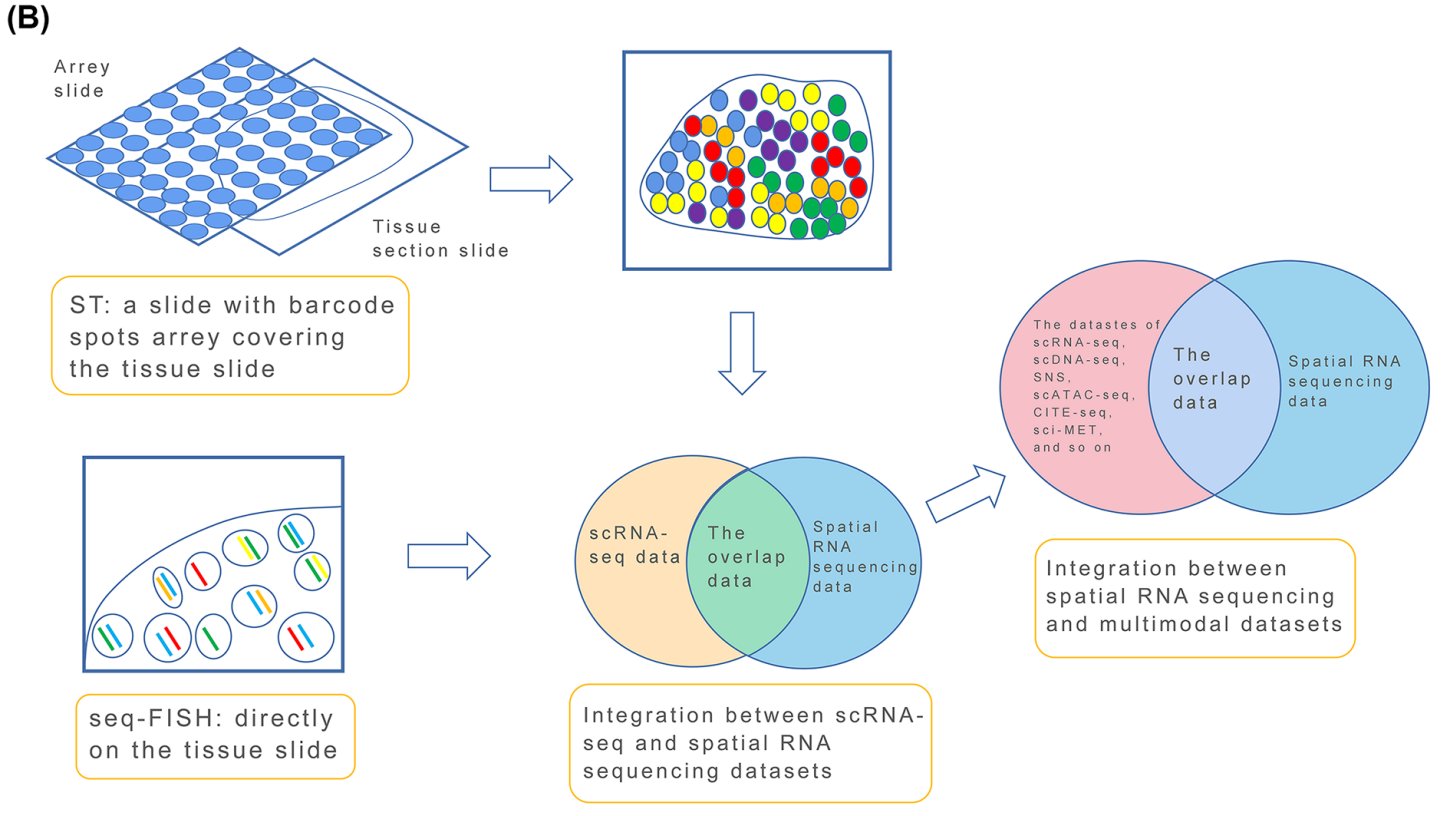
A flow chart shows a sample explanation of seq-FISH and ST spatial RNA sequencing The difference of *in situ* and ST spatial RNA sequencing (**B**).

However, the detection range of *in situ* sequencing methods is often targeted at selected tissue sections, and it is difficult to detect whole tissues, especially those tissue areas that are difficult to target [49] (Figures 2 and 3).

### Spatial transcriptomic sequencing in whole tissue sections

To sequence a large number of RNAs in whole tissue sections, Patrick et al. developed a spatial transchromic (ST) [50] method, by designing a spot array glass slide with spatial bar codes and reverse transcriptase, which was placed on a tissue section, aligned with the position of the tissue section covering the whole tissue section, then synthesized cDNA in the tissue section could be captured in the spot on the array (Figures 2 and 3).

ST is therefore a spatial version of *in situ* FISH of a whole tissue section. All mRNA information in the tissue is collected on a slide at one time. According to the cDNA collected in the spot on the slide, it is possible to determine what transcriptional information exists in the corresponding tissue section location, and to a large extent, the transcriptional information, cell positions, cell types, and the interaction within all tissues can be determined together.

Although ST has improved the integrity of transcriptional information of tissue sections, resolution of a single cell was also reduced [50]. This depended on the size of the spot. The larger the diameter of the spot, the more transcriptional information it collects, and the lower the accuracy of each transcriptional information detected. The spot diameter of ST is 100 µm, and subsequent technology continuously reduced the spot diameter to improve the resolution of the transcriptome in a single cell. For example, Slide-seq (10 µm) [51], high-definition spatial transcriptomics (2 µm) [52], and the Visium platform (55 µm) released by 10× Genomics (https://pages.10xgenomics.com/rs/446-PBO-704/images/10x_BR060_Inside_Visium_Spatial_Technology.pdf) [16] are widely used platforms in cancer research.

We have summarized some applications of spatial transcriptome sequencing methods in tumors (Table 1). It was found that there were significant differences in gene expressions between the central region of tumors and the surrounding adjacent regions. For example, the microenvironment of the central region was mainly composed of stromal cells that expressed aerobic respiration and metabolic pathways, and the surrounding regions were mainly related to the expression of inflammation and immune response [53,54]. Furthermore, oxidative metabolism and lipid metabolism localized in the tumor tissues are different from those in normal tissues, thus, the regulation of cells involved in tumor metabolism could reflect the progress of tumors [55,56].

**Table 1:** The applications of ST in cancer studies

Author	Sample	Method	Main outcome
Thrane Ket et al. [53]	Stage III cutaneous malignant melanoma	ST	In lymphatic cancer metastasis, the expressions of lymphocyte related genes are different between the tissues near cancer and far from the cancer.
Berglund E et al. [54]	Prostate cancer	ST	Different inflammation related genes were expressed in the central region, peripheral region, and adjacent region of the tumor to show different immune microenvironment regions.
Sun H et al. [55]	Pancreatic ductal adenocarcinomas	ST (Visium)	By limiting the oxygen content in the TME region, to predicted the change of gene expressions in tumor tissues.
Lv J et al. [56]	Invasive micropapillary carcinoma of the breast	ST (Visium)	Different regions of the TME exist in different metabolic pathways of tumor related mutant genes.
Yue-Fan Wang et al. [61]	Hepatocellular carcinoma	ST (Visium)	M2 macrophages expressing CCL15 related to the immunosuppressive microenvironment of the core regions of cancer tissues.
Hiroki Murai et al. [57]	Nonviral hepatocellular carcinoma	ST (Visium)	M2 macrophages and CAF were very close to the exhausted CD8+T cells in cancer tissues.
Miranda V Hunter et al. [64]	Melanoma	ST (Visium)	Found the “interface” between tumor and the TME, which consisted of tumors and cells in the TME. Ciliated proteins produced by cells in the TME were enriched in this interface.
Lee-Ann Van de Velde et al. [58]	Neuroblastoma	ST (Visium)	Myeloid cells with metabolizing arginase-1 and CD4+T cells related to tumors were found in the parenchymal region of the tumor.
Juntaro Yamasaki et al. [62]	Gastric cancer	ST (Visium)	Found a group of stromal cells before tumor invasion, which expressed genes related to hypoxia signaling, angiogenesis, and cell migration.
Jingjing Qi et al. [63]	Colorectal cancer	ST (Visium)	Found FAP+ fibroblast and SPP1+ macrophage co-localization, to promote immune repulsive connective tissue hyperplasia and limitation of the infiltration of T cells.

In addition, more studies of ST of the TME have shown that tumor-related stroma cells in the TME are mainly cancer-associated fibroblasts (CAFs). However, myeloid cells, which are mainly tumor-associated macrophages (TAMs), also shared the same locations with tumors. It was found that M2 macrophages, CAFs, and some T lymphocytes were always located in the center of the tumor, and these T lymphocytes were identified as exhausted CD8+T cells [57] and unconventional CD4+T cells [58]. Exhausted CD8+T cells are often found in cancers, which are a type of T cell lacking effector functions [59], and the unconventional CD4+T cells are a type of T cell subtype similar to regulatory T cells [60], which are related to the immunosuppressive and tissue repair systems [58,61]. These two cell types are very close to the localization of M2 macrophages and CAFs, thus they might be regulated by these tumor-related stromal and myeloid cells, resulting in loss of their anti-tumor functions to help tumor cells escape immune response. These studies also revealed the localization of metabolic pathways, which were related to the formation, metastasis, and inhibitory immune system of tumors, and between tumors and these tumor-related stromal cells and myeloid cells [62,63].

Metastatic tumor cells are therefore often accompanied by changes of cells in the TME, so there is a close relationship between tumors and the TME. A study of melanomas and the ST has also shown an interface between tumor cells and cells within the TME [64]. These results indicate that although the cells within the TME are close together with tumor cells, they are not malignant cells, but the two cell types are independent and interdependent, and cell-cell communication links the two groups into a whole system.

### The limitations of spatial transcriptome sequencing methods

High resolution, but lack of tissue localization, are the characteristics of *in situ* spatial sequencing. In contrast, ST with a large-scale sequencing advantage has reduced accuracy of single cell resolution. Hence, how to improve the single cell resolution of ST is the next goal of high-throughput spatial sequencing technologies. There are many ways to improve the resolution. One method involves continuously reducing the diameter of the spot from the abovementioned sequencing methods, and another is to improve the clarity of the imaging technology. Deconvolution is a mathematical algorithm that eliminates previous filtering, and reflects the original clarity of the image using imaging technology. Many imaging technologies therefore design the deconvolution algorithm to achieve high-definition imaging and restore the original image in tissue slices as much as possible.

We have summarized some research methods used to improve ST imaging during the past 2 years, basically involving deconvolution software designed to evaluate the localization of transcriptome expression in ST data through calculations, including SpaGCN [65], MULTILAYER [66], STARCH [67], SPARK-X [68], DeepSpaCE [69], spatialGE [70], MISTy [71], and SpotClean [72]. These methods are mainly aimed at rare cell types existing in complex and multi-level tissue regions that may not be detected by ST, which is equivalent to deep *in situ* sequencing of some key areas in the whole tissue section.

These techniques first detect the transcriptional information in cells, and then determine cell types using other information. Most cell types can be determined using this approach. However, some cell subtypes may share the same transcriptional information with other subtypes, but they are in different functional states.

For example, the previously mentioned CAFs, which are normal fibroblasts in the overactivated TME, are constantly inducing remodeling of the structure and function of the extracellular matrix, followed by promotion of tumor metastasis [28]. For CAFs, we can only identify this cell type by detecting its metabolites and the location in tissue using ST. To a certain extent, this may limit the accuracy of cell type identification. However, by combing the scRNA-seq with spatial transcriptomics sequencing, it is possible to directly map the high resolution of single cell data in tissue sections.

*In situ* spatial RNA sequencing has already shown higher resolution of a single cell than does ST, so when combined with scRNA-seq, it may facilitate characterization of rare cell types. For example, by combining the data of smFISH and scRNA-seq, Massalha et al. [73], in cholangiocarcinoma liver metastases, developed a cellular map of the liver TME, which included different expressions of cell subsets and genes between malignant and non-malignant regions, as well as a stromal cell type related to recurrent tumors, by constructing ligand-receptor interactions.

However, this approach is still limited to specific parts of tissues. We focus next on methods of combining scRNA-seq and ST, to directly map the data of cell types in whole tissue sections.

### The combination of ST and scRNA-seq

The third way to improve resolution is to combine scRNA-seq with ST. The objective of scRNA-seq is to identify cell types, which also provides high resolution of single cells. The combination of these two methods could directly identify cell phenotypes in tissue sections, rather than first detecting transcriptional information in cells, then identifying the cell types; after identifying the cell type, we should be able to locate them in the tissue.

In addition, using scRNA-seq, it may be possible to identify different transcriptional information in the same cell type, for example, distinguishing initial tumor stem cells, progeny tumor cells, and metastatic tumor cells, and then determining the different stages of tumor progression. It may be possible to identify similar transcripts in different cell types, for example, the tumor cells could transform to free mesenchymal cells during the EMT. It may also be possible to show that a cell may interact with adjacent cells to produce mixed transcriptional information; for example, tumor cells may promote their own development by encoding specific metabolites that can induce abnormal functions of stromal and immune cells [74].

Like the difference between NGS and scRNA-seq, ST using NGS can be used to identify and locate the main cell types in tissue sections, but it cannot identify the specific subtypes without using further technology. The combination of ST and scRNA-seq can therefore ensure the high resolution of single cells and show their spatial localizations (Figures 2 and 3).

### Integrating methods

We will review the development of integrating methods. Several deconvolution methods have been used for bulk RNA-seq data to analyze the signals of cell types [75]. Subsequently, Baron et al. for the first time developed BSEQ-sc by using scRNA-seq data [76].

However, these deconvolution methods were based on pre-selected cell marker genes, which were common characteristics of most cell types, but which might omit some rare specific gene expressions, differential gene expressions between individuals, and random gene expressions of the inner cells [77], so analyses of cell types were limited.

Investigators have developed deconvolution methods by using datasets of scRNA-seq to replace marker gene screening. For example, Wang et al. developed multidisciplinary single cell deconvolution, using scRNA-seq datasets instead of marker gene screening to evaluate cell types and proportions in bulk RNA-seq [77]. Butler et al. developed an R package [78] using Seurat software-a computational strategy to identify the cellular localization by integrating scRNA-seq data with *in situ* spatial RNA sequencing [79], by analyzing the overlapping degree of common gene expressions in data from different sources. The higher the degree, the more accurate the determination of a certain cell type. Jew et al. developed Bisque and expanded scRNA-seq datasets, resulting in more refined and diverse cell types identified [80]. Giladi et al. developed PIC-seq by combining physically interacting cells (PIC) with scRNA-seq, which identified the types of these cells, and also evaluated the interaction relationship between them [81] (Figure 1).

Subsequently, investigators have used scRNA-seq datasets to visualize the deconvolution calculation method of cell types in bulk RNA-seq sequencing data with ST. ST itself provides the location of bulk RNA sequencing information in tissue, then the data of bulk RNA sequencing is replaced by scRNA-seq datasets.

We have listed some cancer studies using ST-scRNA-seq, and found that most deconvolution algorithms of ST-scRNA-seq were able to select the gene expressions with high degrees of overlaps, to identify cell types and positions on tissue sections by comparing transcriptomic sequencing information between ST and scRNA-seq datasets (Table 2).

**Table 2:** The applications of the integration methods between ST and scRNA-seq in cancers

Author	Sample	Integrating method	Main outcome
Dylan et al. [89]	High-grade serous ovarian carcinoma	RCTD [90]	Predicting the effect of NACT treatment using cell types and their locations that were involved in EMT pathways.
Andersson et al. [86]	HER2-positive breast tumors	Stereoscope [85]	A tertiary lymphoid structure was found co-localized with tumor tissues, which produced a type I interferon response signal pathway.
Andrew et al. [82]	Cutaneous squamous cell carcinoma	The Seurat R package [78]	Found a subgroup of tumors near the vascular endothelial cells, co-localized with CAFs and inhibitory immune cell populations.
Wu et al. [87]	Colorectal Cancer Liver Metastasis	SCDC [88]	Predicting the effect of NACT treatment using subtypes of macrophages and their metabolic signal pathways of metastatic cancer tissues.
Moncada et al. [49]	Pancreatic ductal adenocarcinomas	MIA [49]	Inflammatory fibroblast populations were found co-localized within tumor regions, and where they expressed genes coding for inhibitory immune responses.
Gouin et al. [83]	Muscle-invasive bladder cancer	The seurat R package	Predicting anti-PD-1 therapy in epithelial cells with CDH12, which were related to the production of exhausted T cells.
Vidhya et al. [93]	Glioblastoma	Spotlight [94]	Found the HMOX1+ myeloid cells localized in the mesenchymal tumor area, which released IL-10 to drive T cell exhaustion.
Wenqin et al. [84]	Medulloblastoma	The seurat R package	Found that differentiation of transformed granular neural progenitor cells in medulloblastomas was significantly inhibited, which was different from normal developing cells.
Youjin et al. [91]	colon adenocarcinoma	XYZeq [91]	Found different cell types of tumor-associated mesenchymal stem cells (MSCs), and some expressions of tumor suppressor genes in the local region of the tumor.
Sunny et al. [92]	Recurrent breast cancer	SCSubtype [92]	Found PD-L1/PD-L2+ macrophages and three different mesenchymal cells in recurrent tumors. Constructed nine types of breast cancer atlases.

Using the previously mentioned Seurat R package, Andrew et al. [82] identified a subgroup of tumors near vascular endothelial cells, which co-localized with CAFs and inhibitory immune cell populations, during a study of cutaneous squamous cell carcinomas. Gouin et al. [83], in a study of muscle-invasive bladder cancers, reported that epithelial cells, with CDH12 expressing PD-L1/2, co-localized with exhausted T cells, with the use of anti-PD-1 immune therapy, showing a good effect. In a study of medulloblastomas, Luo et al. [84] reported that compared with normal developing cells, the differentiation trend of transformed granular neural progenitor cells was significantly weakened.

Similar to the Seurat R package, from the study of HER2-positive breast tumors, there was also a probabilistic model-based method developed called stereoscopy [85]. Andersson et al. [86] found a tertiary lymphoid structure co-localized with tumor tissues, which produced a type I interferon response signal. Wu et al. [87], using the SCDC [88] method in a study of colorectal cancer liver metastasis, found different macrophage subsets and their metabolic signal pathways existed in the TME regions of primary and metastatic cancer tissues.

On the basis of expanding the scRNA-seq dataset, other methods have focused on a more thorough analysis of cell subtypes, to get better consistency and matching degrees of cell types, using multiple platforms and an interdisciplinary approach (Table 2).

Cable et al. [89] used the Robust Cell Type Decomposition method [90], which is a kind of integration method, used to show that the cell types and localizations involved in EMT pathways were changed before/after neoadjuvant chemotherapy (NACT) treatment in high-grade serous ovarian carcinomas. Moncada et al., using a method called multimodal intersection analysis (MIA) [49], reported that inflammatory fibroblast populations were co-localized within tumor regions, and expressed stress response genes in pancreatic ductal adenocarcinomas. Lee et al. developed XYZeq [91], a workflow procedure that encoded spatial metadata into scRNA-seq libraries, and used it in the study of colon adenocarcinomas. It was found that there existed different tumor-associated mesenchymal stem cells during tumor formation, and during this process, there also existed different suppressor genes. Wu et al. developed SCSubtype [92], which was used in the study of recurrent tumors in breast cancers. They identified a new type of macrophage that expressed PD-L1/PD-L2. Furthermore, the mesenchymal cells showed different functions and cell surface protein expressions during differentiation in three major lineages and the whole transcript map of breast cancers. In a study of glioblastomas by Ravi et al. [93], using Spotlight [94], they reported that HMOX1+ myeloid cells releasing IL-10 were spatially located in the mesenchymal tumor area, which could induce exhausted T cells, thereby promoting an immunosuppressive TME. In addition, there is another method called spatialDWLS [95], which is mainly focused on the identity of the cell types in the coexistence boundary. Although it has not been reported to be used for cancer studies, it may be a good method to distinguish tumor cells and the cells in the TME (Table 2).

The combination of ST and scRNA-seq has shown promise in the high resolution of a single cell. However, there are still limitations. ST and scRNA-seq are two independent sequencing methods, so differences in data of the different cell types may exist. Noise is also an inevitable problem of sequencing technologies. Thus, the mismatch between them may lead to wrong mapping regions, and provide false location signals [96].

Therefore, the matching degree of different sequencing platforms is a key factor of integration. The subsequent deconvolution methods basically focus on constantly updating and expanding the sequencing datasets of scRNA-seq, so that it can provide more matching cell types.

### New deconvolution integrating methods in 2022

We next summarize new deconvolution integrating methods reported in 2022. Some methods continued and optimized previous technologies, and improved the detection of cell subtypes in more detail. For example, deconvoluting spatial transcriptomics data using graph-based convolutional networks (DSTG) [97] used CellTrek, a method that detects the topological patterns of different cell types and cell states [98]. CellDART, a method mainly devoted to neural networks [90], and DIALOGUE, a method mainly devoted to identifying multicellular programs among cell–cell interactions [99], and conditional autoregressive-based deconvolution (CARD) [100], have also been used (Table 3).

**Table 3:** Some new deconvolution integrating methods in cancers during 2022

Author	Sample	Integrating method	Main outcome
Ying et al. [74]	Pancreatic cancer	CARD	A variety of cell types and molecular markers were identified, which had clear spatial localizations and which defined the progression, heterogeneity, and regionalization of pancreatic cancer.
Runmin et al. [98]	Ductal carcinoma	CellTrek	Identified tumor subclones and the specific T cell status near the tumor area.
Jerby-Arnon et al. [100]	Lung cancer	DIALOGUE	Found the multicellular programs (MCPs) that were involved in immune activation, tissue remodeling, and cancer immunotherapeutic resistance.
Qianqian et al. [97]	Pancreatic cancer	DSTG	Achieved high level segmentation and revealed the spatial structure of cell heterogeneities in tissues.
Edward et al. [102]	Melanoma, invasive ductal carcinoma and ovarian adenocarcinoma	BayesSpace	Identified tissue structure at the original resolution and transcriptional heterogeneity, and restored, to a large extent, the neighborhood structure of cell types.
Yi et al. [103]	Colorectal cancer	SC-MEB	Compared with BayesSpace, SC-MEB showed a better ability to separate clusters.
Yusong et al. [106]	Pancreatic ductal adenocarcinoma and high-grade serous ovarian cancer	SPCS	Evaluation of combing two factors (ST and scRNA seq) facilitated smoothing the noise and preventing the loss of some important events.

Other methods are based on Bayesian theory, which is a mathematical framework that models reasoning and decision-making under uncertain conditions [101]. These probability model algorithms include BayesSpace [102], SC-MEB [103], and Cell2location [104]. Most of them are used to detect complex cellular components such as brain nerve cells and tumor cells. Because of the continuous expansion of scRNA-seq datasets, these technologies can simulate and predict the localization of different cell types in tissues without the need for experiments on solid tissue components. Therefore, these methods can integrate ST and scRNA-seq data.

Among them, CARD developed by Ying et al. [74] is an interesting method. One of its main features is the clear display of each segregation between different cells, which is the best resolution using this method. These segregations could be presented from malignant and non-malignant regions, to subregions of malignant regions, and even regions that are different between the early and late stages of tumor cell types. The second feature involves finding cell subpopulations that may not be detected by other methods with extremely high resolution. As the authors mentioned, acinar cells were mainly enriched in the areas of normal pancreatic tissues in the study of pancreatic ductal carcinomas, while other methods were unable to adequately localize the main enrichment sites of such cells. The third feature involves locating cell types in different subregions at the same time. These advantages of CARD may provide additional and more accurate information among cellular interactions during the dynamic process of tumor metastasis.

Compared with the above methods, which mainly depend on a large cell population to select high matching degrees of cell types, Missarova et al. developed geneBasis to filter-out the generally expressed genes, and then identified cell types with high specificity and sensitivity. The method mainly focuses on the characterization of rare cell types [105].

In addition to the above methods, which involve expansions of scRNA seq datasets and optimization of integrating algorithms, to improve the imaging resolution, Liu et al. proposed a two-factor smoothing technique involving ST. This method improved the previous one-factor smoothing technique, added spatial sequencing as another factor, so it also used spatial and pattern combined smoothing, which aimed to decrease the noise and dropout events caused by multiple path sequencing [106] (Table 3).

In general, the integration of ST and scRNA-seq datasets to improve resolution of single cells in tissue sections is the main direction of development of spatial transcriptome sequencing methods.

### Future prospects of spatial transcriptome sequencing methodology involving multimodal data analysis, including multi-dimension, multiomics, and interdisciplinary approaches

As the integrating methods of scRNA-seq data on ST become more accurate, investigators are no longer limited in using transcriptomics datasets, but can integrate the multimodal datasets into spatial sequencing, such as genomics, epigenetics, chromatin omics, and proteomics. Thus, more comprehensive cell information in tissues can be displayed using spatial sequencing technology.

These multimodal data methods include the following. Biancalani et al. developed Tangram [107], which integrated many kinds of RNA-seq datasets, like MERFISH, STARmap, smFISH, and sc/snRNA-seq. Jain et al. developed an algorithm for the dimensionality reduction and integration of multiple datasets, called MultiMAP [108], and Veltenjiang et al. developed MEFISTO [109], a multimodal calculation framework based on factor analysis [110]. Vickovic et al. developed spatial multiomics (SM-Omics), as a fully automated, high-throughput platform [111]. These three approaches are major methods for analyzing the combination of multiomics datasets, such as scRNA-seq, scATAC-seq, sci-MET, CITE-seq, and ST datasets (Table 4).

**Table 4:** The methods of combining the multimodal datasets and spatial transcriptomics sequencing

Authors	Sample	Integrating method	Main outcome
Combined with ST			
Mika et al. [108]	Human thymus dataset	MultiMAP	Revealed transcription factor expression and binding site accessibility of T-cell differentiation.
Britta et al. [109]	Mouse gastrula	MEFISTO	By considering the spatio-temporal dependencies of samples, the method combined the continuous covariate among different samples, and continuously and dynamically detected the differentiation trajectory of organisms.
Vickovic et al. [111]	Mouse brain, spleen and colorectal cancer model	SM-Omics	An automated sequencing method mainly for spatial antibody-based multiplex protein detection.
Biancalani et al. [107]	Mouse brain	Tangram	A method combining all RNA datasets with anatomical atlases.
Combined with in situ spatial sequencing			
Chee-Huat Linus Eng et al. [112]	Mouse brain	seqFISH+	Revealed the mRNA localizations of subcellular structures and the ligand-receptor pairs across neighboring cells.
Vu et al. [113]	Colorectal cancer and melanoma	MOSAICA	A multiomics method for mRNA and protein sequencing, which showed the ability of multiplexing scalability.
Park et al. [114]	Mouse brain	SSAM	A robust cell segmentation-free computational framework for identifying cell-types and tissue domains in two- and three-dimension.

Except for the combination of ST, there are many methods integrating multimodal data with *in situ* spatial transcriptomics sequencing. For example, in the early years, Eng et al. developed sequential fluorescence *in situ* hybridization, which was able to identify cell subtypes and receptor ligand pairs between adjacent cells by detecting multimodal signals of chromatin and gene expressions [112]. Vu et al. developed the Multi Omic Single-scan Assay with Integrated Combinatorial Analysis, to detect mRNA and proteins at the same time, and it has been verified in colorectal cancers and melanoma datasets [113]. Park et al. developed Spot-based Spatial cell-type Analysis by Multidimensional mRNA density estimation (SSAM), which is a robust cell free segmentation computing framework, presenting cell types and tissue structures from two- and three-dimensional perspectives [114] (Table 4).

It can be seen that whether it is *in situ* transcriptomic sequencing or ST, or the combination of the two with other datasets, the imaging algorithms are similar, which aim to restore the original spatial location of different cell types, tissue morphologies, and structures in tissue sections as accurately as possible. Although there are few studies using multimodal data analysis methods, they can result in original information of tumor tissues, to provide a reliable basis for the implementation of effective therapies.

## Discussion

In general, sequencing methods have experienced an unprecedented era of rapid development, from sequencing of gene fragments to sequencing of all genetic materials in the whole cell and even whole tissues. Current sequencing technology has been extended to an interdisciplinary analysis model that integrates multiple omics, multiple data, and multiple dimensions.

This has provided many advantages. It greatly improves all cellular and non-cellular information in tissues, and visually displays as much microscopic information as possible. Using a tissue slide, it is possible to identify a certain cell type from multiple perspectives, and collect relevant information about this type of cell. The resolution of cells has been improved from multiple perspectives, and many rare cell phenotypes that were missed by single sequencing technology were found. This expanded cell datasets and increased the number of targets that can be used for new therapies.

However, there are still limitations. Due to the IHT of tumors, more unknown tumor phenotypes may appear in the future, whereas current multimodal datasets are based on the statistics of known experimental research structures. Therefore, while studying new methods, we need to constantly update the information of clinical tumor specimens to keep up with changes of tumor evolution.

The main purpose of spatial sequencing is to visualize the original data in tissue slices. Although multimodal spatial sequencing methods can analyze the information of tissue slides from various angles, these data also depend on tissue samples. Without tissue slides as the main body, these datasets merely comprise data that cannot be used. For tumor metastasis and the complex TME, this is a dynamic process. A tissue slide can only analyze limited information, but cannot track and record a dynamic process. In addition, a mouse model can reflect the evolution of tumors but may differ from human tumors.

The production of sequencing technology ultimately provides information for the transition to clinical medicine. So how do we combine experimental methods with clinical applications? By identifying changes of cell types in tissue sections, we can predict tumor stages and the therapeutic effect for patients. Then, tissue samples at different stages can be selected before and after treatment, and spatial sequencing could be conducted to identify changes of tumor cells and cell types in the TME. It is also necessary to conduct long-term and continuous studies on the same patient or a class of patients with similar diseases. Previous studies have also used single sequencing techniques such as NGS and scRNA-seq to evaluate the therapeutic effect and resistance of patients [115,116].

However, these studies can only predict the treatment effect involving changes of certain gene expressions and cell types, and these predictions only reflect the data from the heat map, but spatial sequencing technology can directly map the data into tissue samples, which is equivalent to indicating the expression information of the tumor related genome, transcriptome, chromatin, protein, and DNA methylation in tissue samples at one time, so that we can intuitively observe these microscopic molecular changes when looking at the patient’s pathological sections. Thus, the accuracy of evaluation is improved to a certain extent. However, this method is more suitable for tissues and organs with obvious solid tumors. For tumor cells with systemic metastasis through the blood, SCS is still best for liquid biopsies [117].

Overall, the use of spatial sequencing technology will promote the development of a personalized and accurate targeted treatment system for patients, to improve the survival of patients with tumor metastasis.

## Data Availability

All data generated or analyzed during this study are included in this published article.

## Abbreviations

CARD: conditional autoregressive-based deconvolution
EMT: epithelial–mesenchymal transformation
FISH: fluorescence *in situ* hybridization
ISS: *in situ* sequencing
LncRNA: long-chain noncoding RNA
MCP: multicellular program
MIA: multimodal intersection analysis
NACT: neoadjuvant chemotherapy
PIC: physically interacting cells
SSAM: Spot-based Spatial cell-type Analysis by Multidimensional mRNA density estimation
TAM: tumor-associated macrophage
TME: tumor microenvironment
